# Identification of potent maturation inhibitors against HIV-1 clade C

**DOI:** 10.1038/srep27403

**Published:** 2016-06-06

**Authors:** Uddhav Timilsina, Dibya Ghimire, Bivek Timalsina, Theodore J. Nitz, Carl T. Wild, Eric O. Freed, Ritu Gaur

**Affiliations:** 1 Faculty of Life Sciences and Biotechnology, South Asian University, New Delhi 110021, India; 2 DFH Pharma, Gaithersburg, MD 20886, USA; 3 Virus-Cell Interaction Section, HIV Dynamics and Replication Program, Center for Cancer Research, National Cancer Institute, MD 21702-1201, USA

## Abstract

Antiretroviral therapy has led to a profound improvement in the clinical care of HIV-infected patients. However, drug tolerability and the evolution of drug resistance have limited treatment options for many patients. Maturation inhibitors are a new class of antiretroviral agents for treatment of HIV-1. They act by interfering with the maturation of the virus by blocking the last step in Gag processing: the cleavage of the capsid-spacer peptide 1 (CA-SP1) intermediate to mature CA by the viral protease (PR). The first-in-class maturation inhibitor bevirimat (BVM) failed against a subset of HIV-1 isolates in clinical trials due to polymorphisms present in the CA-SP1 region of the Gag protein. Sequence analysis indicated that these polymorphisms are more common in non-clade B strains of HIV-1 such as HIV-1 clade C. Indeed, BVM was found to be ineffective against HIV-1 clade C molecular clones tested in this study. A number of BVM analogs were synthesized by chemical modifications at the C-28 position to improve its activity. The new BVM analogs displayed potent activity against HIV-1 clade B and C and also reduced infectivity of the virus. This study identifies novel and broadly active BVM analogs that may ultimately demonstrate efficacy in the clinic.

The human immunodeficiency virus type 1 (HIV-1) is a lentivirus that causes acquired immunodeficiency syndrome (AIDS). Four groups of HIV-1 have been identified, of which group M (main) is the most prevalent and is further subdivided into nine subtypes or clades (A–K) based on the genomic sequence^1^. HIV-1 epidemiological surveys show that clade C accounts for 48% of infections globally and is concentrated mainly in southern Africa and India^2^. HIV-1 clade B is found predominantly in developed Western countries and accounts for only 11% of HIV-1 infections world-wide^2^. Most HIV-1 research is focused on clade B; few laboratories focus on the other clades.

There appears to be a high degree of genetic diversity among HIV-1 clades in different geographical regions. This is a consequence of the high mismatch error rate of the HIV-1 reverse transcriptase (RT) enzyme coupled with the absence of an exonuclease proofreading activity. These inherent differences in clades can influence the effectiveness of any antiviral therapy^3^. Therefore, it is important to understand the efficacy of different drug strategies in non-B clades and to develop broadly active drugs, which will benefit patients not responding to currently available therapy.

The FDA has approved more than 25 antiretroviral (ARV) drugs to treat HIV-1 infection^4^. However, owing to the development of viral-drug resistance and adverse effects associated with currently available drug regimens there is a continuous need to explore new drug targets^5^,^6^. In this regard, 3-*0-(3′-3′*-dimethylsuccinyl) betulinic acid (bevirimat or BVM), a maturation inhibitor (MI)^7^, has been reported to be effective against drug-resistant HIV-1 isolates^8^,^9^. Bevirimat has a unique mechanism of action distinct from that of the known ARVs as it specifically inhibits a single cleavage event, the final Gag processing step in which p25 (CA-SP1) is cleaved to p24 (CA) and SP1 resulting in immature and non-infectious virus. This specificity of BVM suggested that HIV-1 Gag, rather than the PR, is the target of compound binding^8^,^10^,^11^,^12^. A second molecule (known as PF-46396) that is structurally distinct from BVM was also shown to block HIV-1 maturation by disrupting CA-SP1 processing^13^,^14^.

BVM was tested in clinical trials for its ability to reduce viral loads in HIV-1-infected patients. Unfortunately, phase IIB clinical trials were halted as the compound demonstrated efficacy in only 50% of treated patients. It was determined that the lack of activity of BVM in some patients was due to polymorphisms present in the SP1 region of Gag, specifically in a “QVT” motif comprising SP1 residues 6–8^15^,^16^. In particular, it was determined using *in vitro* assays that the SP1-V7A polymorphism reduced the sensitivity of HIV-1 clade B to BVM^17^,^18^. Chemical modifications in BVM increased its potency against BVM-resistant HIV-1 isolates^19^,^20^,^21^,^22^. In a recent report by the Freed group a number of BVM derivatives with modifications at the C-28 heteroatom were screened against HIV-1 clade B and the SP1-V7A derivative. These BVM analogs were significantly more potent than the parental BVM against both the wild-type and the SP1-V7A derivative of the clade B clone NL4-3. In addition, one of the analog also showed antiviral activity against a multi-clade panel of HIV-1 isolates^23^.

In this study, we tested the efficacy of BVM and C-28 alkyl amine derivatives of BVM against multiple HIV-1 clade C clones in both biochemical and virological assays. We found that the BVM analogs could effectively block CA-SP1 processing of HIV-1 clade C in contrast to the parental BVM, which was completely inactive against the clade C clones.

## Results

### BVM is ineffective against multiple HIV-1 clade C isolates

**Figure 1:**
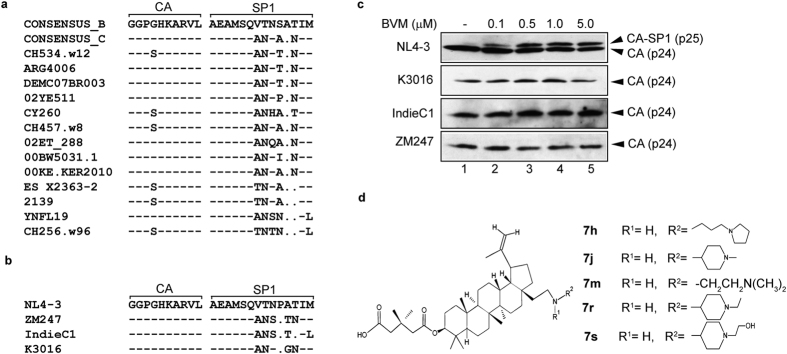
HIV-1 subtype C is resistant to BVM. (**a**) Alignment of amino acid sequences of the CA-SP1 boundary region from multiple HIV-1 clade C isolates compared to consensus HIV-1 clade B sequence. (**b**) Amino acid sequence alignment of the CA-SP1 boundary region for HIV-1 clade B clone NL4-3 and three HIV-1 clade C clones used in this study. Hyphens indicate amino acids identical to the HIV-1 clade B consensus sequence or the NL4-3 sequence (shown at the top); dots indicate gaps. (**c**) CA-SP1 accumulation assay of HIV-1 subtype B and C clones in the presence of BVM. HEK-293T cells were transfected with HIV-1 subtype B clone NL4-3, and subtype C clones K3016, IndieC1 and ZM247. Cells were treated with increasing concentrations of BVM (0.1–5.0 μM) or with DMSO only (lane 1). The virion-associated CA and CA-SP1 was detected by western blotting. Gel images shown here are representative of three independent experiments. (**d**) Shown on the left is the structure of scaffold **7**. Functional groups representing modifications at C-28 position are shown on the right.

It has been reported that BVM is ineffective against a subset of HIV-1 clade B isolates with polymorphisms in Gag, specifically the QVT motif in the SP1 region^15^,^16^. We aligned the CA-SP1 sequence of HIV-1 clade B with multiple HIV-1 clade C clones compiled in Los Alamos HIV-1 sequence database (http://www.hiv.lanl.gov/content/index) and found that these polymorphisms are naturally present in HIV-1 clade C (Fig. 1a,b). HIV-1 clade C infections represent nearly 50% of all HIV-1 infections worldwide^2^; hence for an antiviral drug to be broadly effective against all HIV-1 clades, it is imperative to test its efficacy against HIV-1 clade C. We initially tested the efficacy of BVM against three HIV-1 subtype C molecular clones, K3016, IndieC1 and ZM247. HIV-1 subtype B molecular clone NL4-3 was included as a positive control as it is sensitive to BVM. Briefly, HEK-293T cells were transfected with HIV-1 clade B or C DNA in the absence or presence of increasing concentrations of BVM (0.1–5.0 μM) as described in Methods. Viral supernatants were pelleted and separated on SDS-PAGE followed by western blotting using HIV-IgG antibody. HIV-1 clade B clone NL4-3 showed a dose-dependent accumulation of p25 in the presence of increasing concentrations of BVM confirming previous report^14^, whereas all the HIV-1 clade C molecular clones were found to be resistant to all concentrations of BVM tested (Fig. 1c). These results suggest that Gag polymorphisms in the CA-SP1 region conferred BVM resistance to HIV-1 clade C.

### Novel BVM analogs inhibit HIV-1 clade C maturation

**Figure 2:**
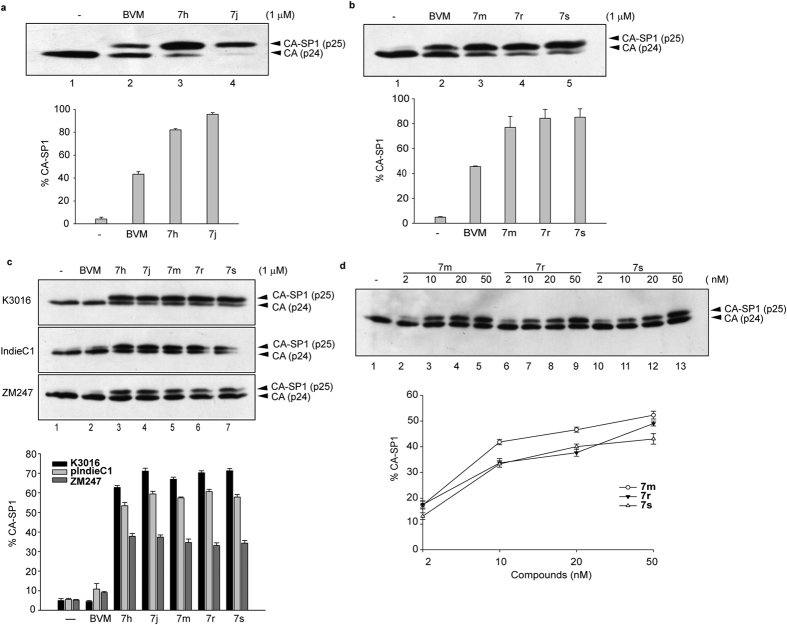
BVM analogs are more potent than BVM. (**a,b**) HEK-293T cells were transfected with the HIV-1 molecular clone NL4-3. Cells were treated with 1.0 μM of BVM, BVM analogs or with DMSO only (lane 1). (**c**) HEK-293T cells were transfected with the HIV-1 molecular clones K3016, IndieC1 and ZM247. Cells were treated with 1.0 μM of the indicated compounds or with DMSO only (lane 1). (**d**) HEK-293T cells were transfected with K3016 in the absence or presence of increasing concentrations (2–50 nM) of BVM analogs **7m**, **7r** and **7s**. The virion-associated CA and CA-SP1 was detected by western blotting. Gel images shown here are representative of three independent experiments. Quantification of % CA-SP1 relative to total CA + CA-SP1 is presented in the graphs. Error bars indicate standard deviations from three independent experiments.

A series of modifications in the C-28 heteroatom of parental BVM were recently reported to be highly active against the wild-type HIV-1 clade B clone NL4-3, a NL4-3 V7A mutant, and a broad panel of primary HIV-1 isolates^23^. We tested the efficacy of five of the most potent BVM analogs (Fig. 1d) against the HIV-1 clade C clones along with HIV-1 clade B clone as control. HEK-293T cells were transfected with NL4-3, K3016, IndieC1 and ZM247 in the absence or presence of BVM and its analogs. Both BVM and its analogs were effective against NL4-3 as reported previously^23^ (Fig. 2a,b). Furthermore, all BVM analogs displayed potent activity against all the three HIV-1 clade C clones relative to the parental BVM, which was ineffective (Fig. 2c). This is evident by increased p25 accumulation observed in the presence of BVM analogs as compared to BVM (Fig. 2c, compare lane 2 with 3–7). A 40–80% accumulation of CA-SP1 intermediate was observed with BVM analogs in all the three HIV-1 clade C clones tested.

We tested the efficacy of three of the BVM analogs (**7m**, **7r** and **7s**) over a range of doses against the HIV-1 clade C clone K3016. As can be seen in Fig. 2d, the analogs induced a dose-dependent accumulation of the CA-SP1 processing intermediate in HIV-1 clade C. We observed nearly 40% activity at 10 nM concentration of the inhibitors. Hence, the BVM analogs are active at very low concentration of the inhibitor.

### BVM analogs reduce infectivity of clade C HIV-1

**Figure 3:**
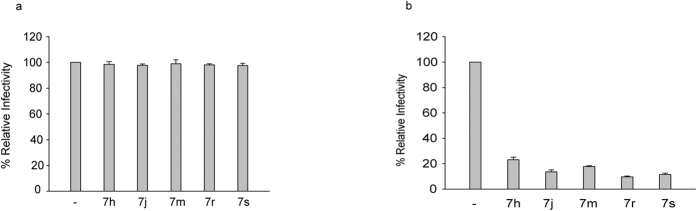
HIV-1 infectivity is diminished by treatment with BVM analogs. (**a**) TZM-bl cells were infected with K3016 WT virus in the presence of 1.0 μM MIs for 1 h. The cells were washed and further incubated for 48 h. (**b**) TZM-bl cells were infected with MI-treated K3016 virus for 48 h. The cells were lysed and assayed for luciferase activity. Quantitative data for levels of infectivity relative to DMSO control treated sample are shown (n = 3).

It is well established that disruption in CA-SP1 processing by HIV PR results in aberrant virion morphology with concomitant loss of infectivity. Because it has been reported that some BVM analogs inhibit both virus maturation and virus entry^20^, we wanted to test whether the MIs inhibited HIV-1 clade C entry or the infectivity of the virus produced in the presence of BVM analogs. We measured the virus infectivity using the TZM-bl cell-based single-round infectivity assay. This assay provides a sensitive and quantitative measure of virus infection as a function of Tat-induced luciferase (Luc) reporter gene expression^24^. Addition of MIs during viral infection did not reduce the infectivity of the virus, indicating that these MIs did not have any effect on the entry of K3016 (Fig. 3a). On the other hand, the infectivity in TZM-bl cells of K3016 virus that was produced from MI-treated cells was reduced by nearly 80–90% as compared to control (Fig. 3b). These results demonstrate that the impaired infectivity of clade C virus induced by these BVM analogs is associated with the defect in CA-SP1 processing that occurs during virus maturation.

### BVM analogs display improved *in vitro* antiviral activity against HIV-1 subtype C relative to BVM

For determination of *in vitro* antiviral activity of BVM analogs, we used spreading HIV-1 infection in the HUT-R5 T-cell line. HUT-R5 cells were infected with K3016 virus and cultured for eight days in the presence of serial dilutions of each compound. Antiviral activity was monitored by reduction in HIV-1 p24 levels in culture supernatants. IC_50_ values for compounds are presented in Table 1. Though the parental compound BVM was ineffective against HIV-1 subtype C (IC_50_ of more than 1,000 nM), the second-generation BVM analogs showed significantly improved antiviral activity in the low-nanomolar range. We also evaluated the cytotoxicity of the BVM analogs in HEK-293T and HUT-R5 cells as described in Methods. The CC_50_ values for these BVM analogs were close to that of the parental compound BVM (Table 1).

## Discussion

Recently, Urano *et al.* reported improved antiviral activity of a set of BVM analogs with C-28 modifications against an HIV-1 clade B clone (NL4-3) and a variant of NL4-3 containing the SP1-V7A polymorphism^23^. In the current study, we extended the earlier analysis by performing biochemical assays to correlate the anti-clade C antiviral activity with a block in CA-SP1 processing. For this analysis, we used three independent clade C clones. The BVM analogs evaluated here were potent in inhibiting the CA-SP1 processing of HIV-1 clade C Gag. Furthermore, these compounds displayed low-nanomolar antiviral activity against HIV-1 clade C with cytotoxicity comparable to that of parental BVM.

Several reports have suggested that C-28-modified BVM analogs inhibit virus entry as well as virus maturation^20^,^25^,^26^. Dang *et al.* also reported synthesis of BVM analogs with C-3 and C-28 modification effective against NL4-3 V7A derivative^21^. The analogs used in our study did not show any effect in inhibiting entry of HIV-1 clade C virus, consistent with the results of Urano *et al.*^23^. The antiviral activity of all the BVM analogs tested correlated well with CA-SP1 accumulation and the virions produced in the presence of the analogs were poorly infectious.

Several studies using ^3^H-labeled BVM and photoaffinity analogs of BVM have indicated that the interactions between the MIs and HIV-1 Gag mainly occur in the CA-SP1 boundary region and the major homology region of CA^10^,^27^,^28^. Indeed, mutations in specific residues within the CA-SP1 region result in loss or reduction in binding^27^,^28^. The lack of activity of BVM against HIV-1 clade C could be due to its inability to interact with Gag. The mechanism that underlies the increase in activity of BVM analogs against HIV-1 clade C is still not clear. But the modifications at the C-28 heteroatom in the parental BVM could lead to tighter binding of the novel BVM analogs to the CA-SP1 region of HIV-1 clade C. Furthermore; in our experiments we observed that the three HIV clade C molecular clones displayed differential sensitivity against the BVM analogs with K3016 being most sensitive. This could be due to inherent changes in the amino acids sequence in the CA-SP1 region amongst the three-clade C molecular clones, which could lead to change in binding affinities of the compounds. Experiments using ^3^H-labeled BVM analogs as well as virus selection experiments in the presence of BVM analogs would help in identifying the putative residues in HIV-1 clade C CA and SP1 that could be interacting with the compounds. It would be interesting to compare the residues in HIV-1 clade B versus clade C Gag that interact with the MIs and relate this to their activity. Ongoing structure-function studies will be highly beneficial in identifying novel and broadly active maturation inhibitors and help in their clinical development as potential ARVs.

## Methods

### Chemical synthesis

BVM derivatives used in the study were synthesized by modifications at the C-28 heteroatom as described previously^23^. Compounds **7h**, **7j**, **7m**, **7r** and **7s** were selected for the present study. All the compounds were dissolved in dimethyl sulfoxide (DMSO) and stored in the dark at −80 °C.

### Plasmids, tissue culture and transfections

HIV-1 clade B molecular clone NL4-3 and clade C clones K3016, ZM247 (a kind gift from Dr. Christina Ochsenbauer, University of Alabama, USA) were used in this study. Both K3016 and ZM247 have been constructed using viral strains from South Africa. The HIV-1 clade C molecular clone, pIndieC1, was a kind gift from Dr. Uday Ranga, JNCASR, India. This clone has been constructed using HIV strain from India. HEK-293T and TZM-bl cells were propagated in Dulbecco’s modified Eagle’s medium (DMEM) containing 10% fetal bovine serum (FBS). HUT-R5 cells were propagated in RPMI-1640 medium supplemented with 10% FBS. For transfections, HEK-293T cells were grown in six well plates to about 80% confluency. Cells were transfected using Lipofectamine 2000 (Invitrogen, USA) following manufacturer’s recommendations. For viral replication assays, HUT-R5 T-cells were transfected using DEAE dextran as described previously^29^.

### Preparation of viral stocks

Virus stocks were prepared by transfecting HEK-293T cells with HIV-1 DNAs (3 μg). 24 h post-transfection, the culture medium was replaced with fresh DMEM and incubated for another 2 h. MIs were maintained in the culture throughout transfection. The culture supernatant was centrifuged at 845 × *g* for 3 min to remove cellular debris. The clarified supernatants were filtered (pore size 0.45 μm filter disc) to remove residual cellular contaminants. For determination of viral infectivity, the unconcentrated virus was used to infect TZM-bl cells. For the CA-SP1 accumulation assay, the virus was pelleted by ultra centrifugation at 210,100 × *g* for 1 h at 4 °C using SW41Ti rotor (Beckman Coulter, USA).

### CA-SP1 accumulation assay

To measure accumulation of CA-SP1, immunoblot analysis of virus-associated proteins was performed. The virus pellet was resuspended in radioimmunoprecipitation assay (RIPA) buffer (50 mM Tris-HCl pH 8.0, 150 mM sodium chloride, 1.0% NP-40, 0.5% sodium deoxycholate, 0.1% SDS) containing 1 × protease inhibitor cocktail (Roche, Germany). The viral lysates were subjected to SDS-polyacrylamide gel electrophoresis (15% gel); proteins were transferred to polyvinylidene difluoride membranes and reacted with HIV-IgG obtained from the NIH AIDS Reagent Program (catalog no. 3957) followed by incubation with HRP-conjugated anti-human secondary antibodies (GE Healthcare, UK). The proteins were visualized by enhanced chemiluminescence (Pierce, USA) and the bands were quantified using ImageJ software (http://imagej.nih.gov/ij/).

### Viral infectivity assays

The virus stocks were normalized for p24 antigen using an HIV-1 p24 Antigen Capture kit (ABL, USA). Equal amounts of virus were used to infect TZM-bl cells (5 × 10^4^/well) in the presence of 20 μg DEAE-dextran per ml in 24 well plate. Single-round infectivity assays were performed as previously described^30^. The luciferase activity in the cell lysates was measured using the Steady-Glo luciferase assay kit (Promega, USA) following manufacturer’s recommendations.

### Antiviral assays

HUT-R5 cells were infected with normalized HIV-1 clade C K3016 virus stocks at 37 °C for 1 h. Cells were then maintained for 8 days in the presence of serial dilutions of MIs. After 8 days, the HIV-1 p24 concentration in the virus supernatants was measured to monitor virus replication. The 50% inhibitory concentrations (IC_50_s) were determined as the concentrations of MI that reduced HIV-1 p24 levels to 50% relative to DMSO-only controls.

### Cytotoxicity assays

Cytotoxicity assays were performed using the CellTitre-Blue Cell Viability Assay kit (Promega, USA) as per manufacturer’s recommendations. HEK-293T and HUT-R5 cell lines were maintained in the presence of serial dilutions of MIs for 4 days and treated with CellTitre-Blue reagent for 4 h at 37 °C. The fluorescent signals were recorded at 530/25_excitation_ and 590/35_emission_ using BioTek microplate reader. The 50% cytotoxicity concentrations (CC_50_s) were determined as the concentrations of MI that reduced the fluorescent signals to 50% relative to DMSO only controls.

## Additional Information

**How to cite this article**: Timilsina, U. *et al.* Identification of potent maturation inhibitors against HIV-1 clade C. *Sci. Rep.* **6**, 27403; doi: 10.1038/srep27403 (2016).

## Figures and Tables

**Table 1:** Antiviral activity against HIV-1 subtype C virus (K3016)* and cytotoxicity# of BVM analogs.

Compound	IC_50_ (nM)	CC_50_ (μM)	
		HUT-R5	HEK-293T
BVM	>1000	>1.0	>5.0
7h	11.3	>1.0	>1.0
7j	5.9	>1.0	>1.0
7m	1.4	>1.0	>1.0
7r	1.5	>1.0	>1.0
7s	1.3	>1.0	>1.0

^*^The IC_50_ values are representative data from two independent experiments.

^#^The CC_50_ values represent mean ± S.E.M. from three independent experiments.
